# The health status of incarcerated people in Canada: a narrative review

**DOI:** 10.3389/fpubh.2026.1902956

**Published:** 2026-09-11

**Authors:** Amanda Slaunwhite, Sidney Hoolsema, Ananya Ogiral, Kate Roth, Sammy Smith, Ashok Krishnamoorthy, Annabel Mead, Ruth Elwood Martin

**Affiliations:** 1 School of Population and Public Health, University of British Columbia, Vancouver, BC, Canada; 2 BC Mental Health and Substance Use Services, Provincial Health Services Authority, Vancouver, BC, Canada; 3 Department of Psychiatry, University of British Columbia, Vancouver, BC, Canada

**Keywords:** Canada, correctional health, health status, incarceration, prison, prison health

## Abstract

**Background:**

People who experience incarceration in Canada have been historically more likely to experience poor health than community-based populations. Although incarcerated people are entitled to health care equivalent to community standards, evidence suggests this is inconsistently achieved in Canada. We conducted a narrative review to synthesize contemporary evidence on health status and health care utilization by incarcerated people in Canada, including changes observed during and after the COVID-19 pandemic.

**Methods:**

We searched MEDLINE, PsycINFO, CINAHL, Scopus, and Web of Science for peer-reviewed literature published between January 1, 2014 and July 22, 2026 on the health of adults incarcerated in Canadian provincial, territorial, and federal correctional centers. Both title/abstract and full-text screening were completed independently by two reviewers, and findings were extracted and synthesized thematically across major health domains.

**Results:**

Across the 167 included articles, incarcerated people in Canada experience significantly higher rates of mental illness, substance use disorders, chronic disease, hepatitis C virus infection, and HIV. Mental health and substance use disorders were highly prevalent and often concurrent. Access to reproductive health care and maternal care in custody was limited compared to community care. Health care continuity was disrupted at admission, transfer, and release from correctional centers. These disparities were closely tied to community-based social determinants of health such as poverty, unstable housing, and systemic racism, and mortality risk was highest during transitions in and out of custody, when the risk of overdose death in the weeks following release was greatest.

**Interpretation:**

Incarceration is an important structural determinant of health in Canada that intersects with community-based social determinants of health, contributing to health inequities. To reduce preventable death among people who experience incarceration in Canada, efforts are required to strengthen continuity of care, evaluate the quality of services in correctional centers, and address the social determinants of health and structural inequities that contribute to premature mortality.

## Introduction

People who are incarcerated in Canada experience pervasive and sustained health inequities. Research has consistently shown that there is a higher prevalence of mental illness, substance use disorders, chronic disease, infectious diseases, and premature mortality among incarcerated people compared to community populations (1, 2). These disparities reflect layered upstream structural determinants of health, including poverty, violence, unstable housing, systemic racism and trauma that can be transmitted across families, communities, and generations (3).

Due to the disproportionately high burden of illness among incarcerated people in Canada and internationally, correctional facilities are a critical setting for health care delivery and public health interventions (4–7). International human rights standards, including the United Nations (UN) Standard Minimum Rules for the Treatment of Prisoners (commonly known as the Mandela Rules), establish that people held in custody must be able to obtain the same standard of health care as anyone else (8). The Mandela Rules also outline that health care should be provided in close alignment with public health systems (8). The World Health Organization (WHO) has similarly emphasized that prison health is an integral part of public health and should be organized and delivered in close collaboration with community health services (9). In Canada, the principle of health care equivalence is broadly recognized; however, correctional health services are organized and delivered across federal, provincial, and territorial jurisdictions with varying degrees of integration with community-based services (10). Transfers between correctional centers and abrupt releases back to community can further interrupt the continuity of care, particularly for treatment of mental health, substance use disorders, and sexually transmitted and blood-borne infections (11–13). The consequences of these interruptions are measurable: maintaining opioid agonist therapy while in provincial custody lowers a person’s likelihood of overdose in the weeks immediately after they return to the community (12), and being moved between facilities or released partway through hepatitis C therapy sharply reduces the chance that a course of treatment is ever finished (11).

Prior literature reviews have documented the need for improved health care services for incarcerated people in Canada but have not captured the evolving landscape of the overdose crisis, the expansion of opioid agonist treatment and associated harm reduction initiatives (14). Furthermore, this research predates the COVID-19 pandemic, which exposed structural vulnerabilities within correctional settings, and coincided with an increase in overdose-related deaths post-release (15).

The objective of this narrative review is to synthesize contemporary peer-reviewed evidence on the health of incarcerated adults in Canada, focusing on the major health domains relevant to public health. The review identifies opportunities and implications for health care operations, policies, and research focused on incarcerated people in Canada.

## Methods

### Design

**Figure 1:**
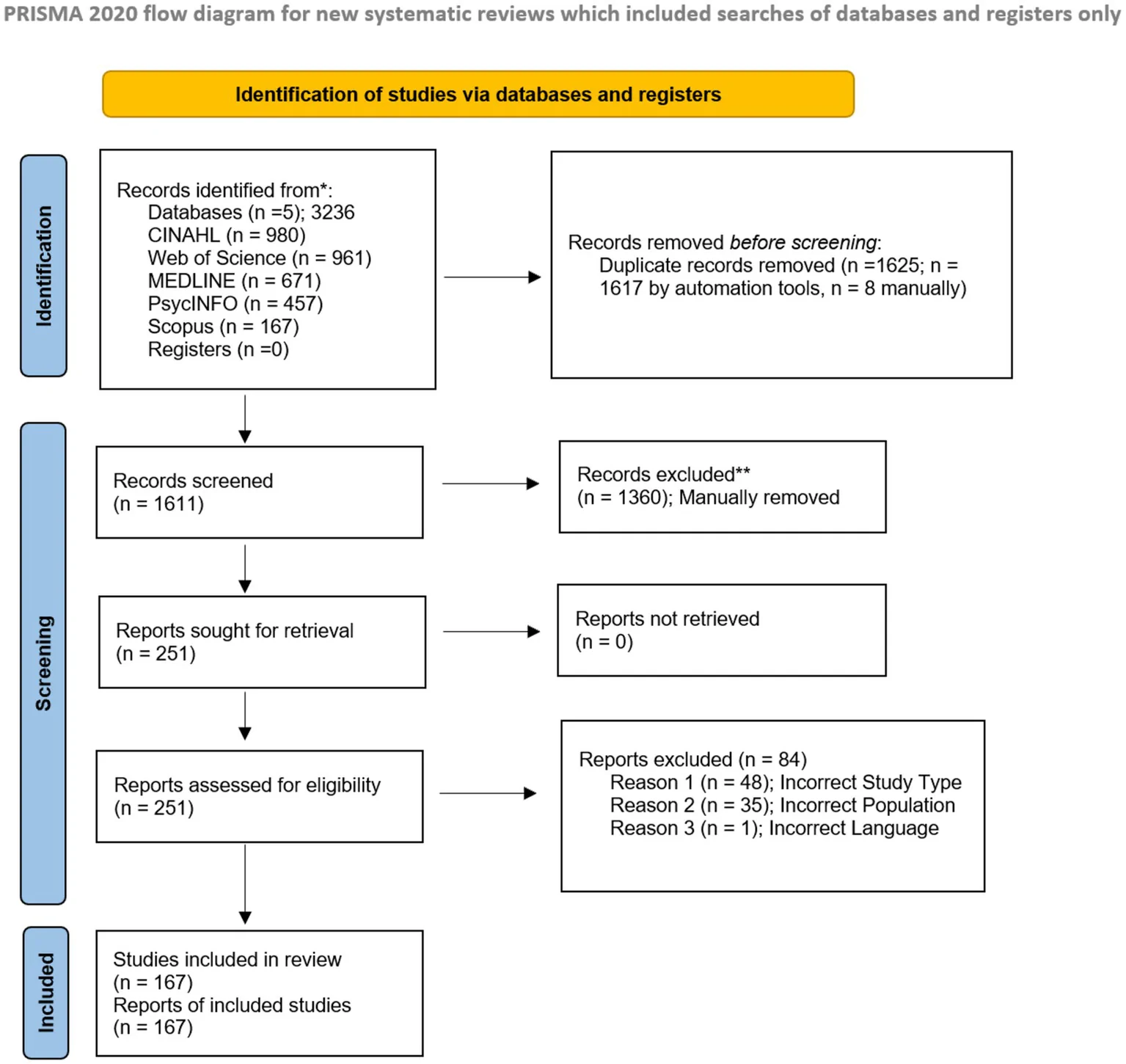
PRISMA 2020 flow charts.

We conducted a comprehensive narrative review of peer-reviewed literature examining the health status of incarcerated adults in Canada, covering the period from January 1, 2014, to July 22, 2026. This timeframe was selected to build upon the previous review by Kouyoumdjian et al. (14), which was completed in 2014. This review was not registered, as PROSPERO does not accept registrations for narrative reviews. Study identification and selection are reported using the PRISMA 2020 flow diagram (Figure 1); the full PRISMA checklist, which is intended for systematic reviews, was not applied.

### Search strategy

A subject area librarian collaborated with two reviewers (SH and SS) to develop a comprehensive search strategy and identify appropriate databases. Searches were conducted in MEDLINE, PsycINFO, CINAHL, Scopus, and Web of Science. In brief, the search combined terms for incarceration (e.g., prison*, incarcerat*, inmate*, correctional*, carceral*) with terms for Canada and its provinces and territories and, where applicable, health-related terms, using both keywords and subject headings (e.g., the MeSH terms). The full search strategy is available in Supplementary material. A small number of relevant Canadian articles published in journals not indexed in these databases were identified through hand-searching and are cited in the narrative synthesis; these are not included in the count of articles retrieved through the database search.

#### Inclusion criteria

Studies were included if they reported research conducted in Canada focused on the health of adults aged 18 years or older who were incarcerated in any Canadian correctional setting, including provincial, territorial and federal correctional centers. Eligible studies were peer-reviewed publications, including commentaries, reviews, and articles where formerly incarcerated people reflected on their health during incarceration.

#### Exclusion criteria

Studies were excluded if they were not conducted in Canada, were not published in English, or were published prior to 2014. We also excluded grey literature, including dissertations, as well as studies that focused exclusively on recidivism, post-incarceration outcomes, or economic analyses. Grey literature was excluded from the search to focus on synthesizing peer-reviewed literature to support the reliability and robustness of our search process. Research conducted in forensic mental health facilities, studies on the health of correctional staff, non-human studies, and studies involving individuals under 18 years of age were also excluded. The full eligibility criteria are summarized in PICOT format in Supplementary Table 1.

### Data extraction and synthesis

All retrieved citations were imported into Covidence, where duplicates were automatically removed. Title and abstract screening was conducted independently by two reviewers (SH and SS), and full-text screening was likewise completed independently by two reviewers (SH and AO). Disagreements at both screening stages were resolved through discussion between the two reviewers, and where consensus could not be reached, the first author (AS) was consulted to reach a final decision.

Thematic analysis was performed by SH and AO through iterative extraction of data, grouping related findings into the major health domains reported below, and refining these categories through discussion until the reviewers reached consensus. For each eligible citation, data were extracted according to study characteristics, study location and study design. Literature classifications were created prospectively and categorized in Excel by the following headings: “Literature Classification,” “Health Factors,” “Study Objective,” “Main findings,” “Status of incarceration at time of study”, “Article type”, “Pre, during or post-COVID”, “Peer Collaboration (yes or no)”.

This study uses a narrative review method, and the quality of individual articles was not formally appraised. Formal critical appraisal instruments are intended to weight or exclude studies contributing to a pooled estimate or grading, neither of which this review produces, and the included literature spans study designs and publication types to which no single instrument applies.

## Results

### Summary of included research

A total of 167 articles met the eligibility criteria (Figure 1). Research often focused on multiple health issues experienced by incarcerated people, particularly women, older adults, Indigenous Peoples, and people with substance use disorder and/or mental illness. Research was concentrated on physical health (*n* = 62), women’s health (*n* = 37), mental health (*n* = 40), substance use (*n* = 28), Indigenous Peoples’ health (*n* = 7), health care utilization (*n* = 20), public health and infection control (*n* = 17), treatment (*n* = 16), mortality (*n* = 13), sexual and reproductive health (*n* = 18), and social determinants of health (*n* = 8). These literature classification categories were not mutually exclusive; articles reporting findings across more than one health domain were counted under each applicable category.

Studies were categorized as federal (*n* = 59), provincial or territorial (*n* = 68), or both federal and provincial (*n* = 17); jurisdiction could not be determined for 23 studies. Provincially, studies were distributed as follows: Ontario (*n* = 42), British Columbia (*n* = 16), Alberta (*n* = 11), Quebec (*n* = 4), Newfoundland and Labrador (*n* = 2), Nova Scotia (*n* = 2), New Brunswick (*n* = 1), Saskatchewan (*n* = 1), and Manitoba (*n* = 1). No studies were set in Prince Edward Island, Yukon, the Northwest Territories, or Nunavut. A further 63 studies were pan-Canadian, drawing on samples spanning multiple provinces or territories, while “Western Canada” (*n* = 3) and “Atlantic Canada” (*n* = 1) were used in place of specific provincial labels. Studies that did not specify a geographical location were classified as “Not Applicable” (*n* = 20).

When categorized by timeline, we found that the majority of the literature examined was published in the pre- COVID-19 era (*n* = 81), followed by literature published during COVID-19 (*n* = 60) and the post-COVID-19 period (*n* = 26). Across all timelines, most synthesized literature was original research (*n* = 126), followed by commentaries (*n* = 15), reviews (*n* = 13), reports (*n* = 12), and “other” (*n* = 1).

Studies were categorized based on participant incarceration status at the time of data collection. The majority of studies involved participants who were incarcerated (*n* = 131). Fourteen studies (*n* = 14) categorized as “not incarcerated” featured post-release participants reflecting on their past incarceration experiences. Finally, “other” (*n* = 22) was used for studies where the incarceration status was undefined. Characteristics of each included study, including article type and research design, population and setting, health domain, and outcomes measured, are provided in Supplementary Table 2.

### Physical health

#### COVID-19 pandemic and vaccination

Correctional centers in Canada were repeatedly identified as high-risk settings for SARS-CoV-2 transmission because of overcrowding, poor ventilation, shared living and unsanitary facilities, and the difficulty of maintaining physical distancing and other infection prevention and control measures (16–18). A seroprevalence study across three Quebec provincial prisons found that 22% of incarcerated men had been infected with SARS-CoV-2, and that seropositivity rose with time spent incarcerated, evidence that the carceral environment itself accelerated transmission, reaching levels several-fold higher than in surrounding communities (19). This pattern held at the population level: among all Canadian congregate settings, correctional facilities recorded the highest COVID-19 outbreak intensity and the largest outbreaks (20), and in Ontario, COVID-19 incidence among incarcerated people consistently exceeded that of the general population, peaking in January 2022 at roughly eleven times the community rate (21). High rates of admission, release, and transfer, particularly in remand settings, further amplified the risk of viral introduction and spread (20). Risk of infection was also elevated for people who were members of a visible minority or who experienced unstable housing and employment (19). Together, these findings echo pre-pandemic evidence that Canadian correctional facilities are prone to respiratory outbreaks under the same structural conditions (22).

During the early pandemic, physical distancing and infection-control measures disrupted the Canadian legal system, including court operations, reducing the number of people held in correctional centers, most notably a 20% reduction in Quebec prisons undertaken to relieve overcrowding (19). This decarceration, combined with infection-control efforts, is thought to have lowered transmission within correctional centers (19). Even so, prevention remained especially difficult for incarcerated people with mental illness or substance use disorders, for whom adherence to infection-control measures was harder to sustain (17).

Beyond acute infection risk, the pandemic also disrupted routine and chronic disease care in custody. Hepatitis C antibody screening in Quebec’s largest provincial prison fell significantly during and after outbreaks and had not recovered to pre-pandemic levels by early 2022, as nursing resources were diverted to SARS-CoV-2 screening and mandatory admission isolation reduced screening opportunities (23). Access to mental health treatment and correctional programming was similarly modified or suspended under prolonged lockdowns (24), and reviews of the pandemic response documented long waits for health services (25).

Vaccine uptake among incarcerated people lagged behind that of the community. Population-level analyses in Ontario found lower COVID-19 vaccine coverage among incarcerated people than in the general population (21). Studies of people in Canadian federal correctional centers identified several factors shaping uptake, including trust in health care, prior receipt of influenza vaccination, and security classification (26–28). Commentary situated vaccination in prisons as a matter of justice and public health (29), while further research highlighted the value of Indigenous, and gender-based approaches to understanding the pandemic’s impact on incarcerated people (18).

Although COVID-19 mortality was lower among incarcerated populations than in the community, the pandemic heightened the risk of overdose death after release (15). Mortality risk was greatest in the period following release, with women experiencing greater overdose risk and men greater COVID-19–related mortality (15).

#### Hepatitis C virus (HCV)

Incarcerated people were identified as a priority population for HCV prevention and elimination due to elevated prevalence, risk of transmission and fragmented access to health care (30, 31). Research found that one in nine Canadians with HCV were incarcerated each year, and women, older adults, people who inject drugs or are tattooed in custody, and people who are incarcerated more than once were at greater risk of HCV infection (30–33). Studies emphasized the need for opt-out HCV screening, rapid treatment initiation, and linkage to community health care services to advance micro-elimination efforts (11, 32). Challenges in providing HCV treatment to incarcerated people were well documented, including inconsistent assessment, delayed treatment initiation, and limited continuity following transfer or release (34, 35). HCV treatment completion rates were the highest among people who completed therapy in correctional centers and substantially lower among people transferred or released mid-treatment, underscoring the importance of treatment continuity post-release (11).

#### HIV

Incarcerated people were also identified as a priority population for HIV testing and treatment due to a similar trifecta of high prevalence, fragmented access to treatment, and risk of infection. Among people with HIV admitted to provincial prison in Ontario, 34.0% received HIV care while in custody and an estimated 52.4% were receiving antiretroviral therapy; in the 6 months following release, 60.1% filled an antiretroviral therapy prescription, compared with 79.6% of people with HIV in the general population (36, 37). In qualitative research, incarcerated women identified stigma, discrimination and fear as major barriers to receiving HIV care (38). Likewise, peer-focused HIV prevention interventions were identified as a promising approach to HIV prevention among incarcerated men (39).

#### Sexually transmitted and blood-borne infections (STBBIs)

Correctional settings were repeatedly identified as underutilized sites for STBBI testing and treatment due to low uptake of optional screening (40). Current research suggests that opt-out STBBI screening increases diagnostic rates, and that universal screening among the incarcerated population could serve as a cost-effective outbreak management strategy. Relying on a traditional opt-in, risk-based approach is a missed opportunity for detection in a population that has higher STBBI rates (41).

A study from an Alberta provincial correctional center examining opt-out STBBI screening at admission found that the opt-out approach resulted in higher diagnostic, positivity, and case-finding rates than the opt-in approach (41). Another study from Newfoundland and Labrador found that sex was a significant predictor of STBBI testing, and that only 72 of 1,611 admissions (4.5%) had any STBBI result on record; 37.5% of those tested returned at least one positive result, and uptake varied nearly six-fold across facilities (from 1.9 to 11.2%) (42). Incarcerated male individuals were less likely to be tested for STBBIs than females (42).

#### Nutrition and weight management

Correctional centers were described as obesogenic environments where poor diet quality and limited physical activity were pervasive (43–45). Two cohort studies found that 73% of federally incarcerated people gained weight during incarceration, with obesity prevalence increasing from 26% at admission to 46% at follow-up (43, 46). Of those who had a normal body mass index (BMI) upon admission, 52% no longer did after being incarcerated for at least 6 months (43). Weight gain was associated with being older, regionality, ethnicity, and longer incarceration (43).

The introduction of “cook-chill” food systems contributed to nutritional inadequacy, weight gain, malnutrition, and perceived degradation of food quality (47–49). Qualitative studies highlighted how limited access to food and reliance on pre-packaged canteen foods contributed to poor nutritional outcomes (50). Recent evidence suggests that social support, including visits and phone contact, may reduce weight gain during incarceration (51).

### Mental health

Mental health issues and diagnosed mental health disorders are much more pervasive among incarcerated people compared to community-based populations in Canada. Research using a validated correctional screening instrument has found that 12 to 14% of incarcerated people in Canada have current, severe symptoms of a psychotic disorder, bipolar disorder, or major depression (52). Linked administrative data from British Columbia provide a direct community comparison: in 2021, 51.1% of people incarcerated in provincial custody had a diagnosed mental illness and 41.0% had co-occurring mental illness and substance use disorder, with substance use disorder present in 58.9% of incarcerated people compared with 3.2% of non-incarcerated people (53). Inconsistences in providing mental health supports between community and custodial settings constrain service delivery, delaying timely care to incarcerated people (54, 55).

Mental health disorders were highly prevalent among incarcerated people, far exceeding rates among community-based populations. Studies documented high rates of depression, anxiety, psychosis, cognitive impairment, and concurrent substance use disorders (2, 52). Women and older incarcerated adults experienced particularly high rates of mental illness, trauma, chronic pain, and disability (56, 57). Segregation or solitary confinement within correctional centers was associated with poorer mental health and an elevated risk of self-harm and suicide (58, 59).

Current research has evaluated the reliability and validity of mental health screening assessments at intake (60). Further investigation is required to examine how these diagnostic thresholds perform across diverse demographics, such as racial minority groups (54). A 2022 study found co-occurring mental illness and substance use disorder in close to one-third (32.0%) of 2017 admissions to custody (61). This marked increase could reflect changing patterns in the presentation of mental disorders upon admission, or availability of supports provided in the community (61). Treatment services within prison may not be equipped to meet the needs of individuals with co-occurring disorders at admission (54, 60, 61). Psychotropic medication use and off-label prescribing raised concerns about the use of pharmacotherapy in the absence of trauma-informed psychological care in correctional centers (62, 63). The challenges of providing psychiatry services in correctional centers was also found to further constrain service delivery (55).

While current literature suggests a temporal link between the duration of pre-sentence detention and symptom severity, research continues to treat mental health disorders as a homogeneous group (52). Because different psychiatric conditions follow distinct trajectories, future research should move beyond treating time as a passive factor and instead examine specific variables that exacerbate symptoms in carceral environments. Additionally, studies should stratify findings to account for differences in sentenced and remanded populations.

### Substance use

Substance use disorders were among the most prevalent health conditions affecting incarcerated populations and frequently co-occurred with mental illness (61, 64). Access to OAT in correctional centers improved in recent years, but there were inconsistencies in OAT access across jurisdictions and facilities, with varying approaches to treatment initiation at intake (65–68). In Ontario provincial prisons, for example, the proportion of incarcerated people prescribed OAT increased from 8.2 to 13.2% between 2015 and 2018, driven largely by an increase in buprenorphine/naloxone prescribing (from 0.8 to 4.8%) (65–68). Harm reduction services, including prison needle exchange programs, were implemented in a small number of federal correctional centers in Canada (69, 70).

### Women’s health

Unmet contraceptive needs, limited access to abortion services, inadequate antenatal care, and disrupted relationships between parents and infants highlighted the discontinuity of gender-specific care (71–74). These gaps reflected not only limited availability of services but also policy constraints and barriers to health-care access within custodial settings, rather than a simple absence of care. In the Canadian context, these shortfalls reflected a combination of policy gaps and barriers to health-care access rather than an explicit prohibition of care: there were no national or provincial policies governing the provision of contraceptive services in correctional facilities, and initiation of contraception remained uncommon despite an unmet contraceptive need approaching 80% among women at risk of unintended pregnancy (71). Obligations to provide gender-specific care at a standard no lower than what community services offer, set out in the *Corrections and Conditional Release Act*, Correctional Service Canada’s health-services directive, and the United Nations Bangkok and Mandela Rules, existed but were inconsistently implemented and appeared to go unmet (75). Access was further constrained by short and often unpredictable periods in custody, high rates of remand, frequent inter-facility transfers, poor coordination with community providers, and the geographic isolation of many women’s facilities from obstetric and reproductive services, which limited the delivery of contraception, antenatal care, and abortion even where eligibility was not in question (72, 75, 76). Because abortion in Canada is publicly funded and not subject to criminal restriction, limited abortion access reflected these service-delivery and logistical constraints rather than legal prohibition.

In Canada, women are one of the fastest-growing prison populations (73) and have distinctly different pathways into prison compared to men (77). In 2020/2021, Indigenous adults accounted for 42% of women in provincial admissions, and 49% of women federal admissions in 2022/2023 (78), despite making up only 4.9% of the general Canadian population (79). Of the Indigenous women incarcerated in federal correctional centers, 97% experienced mental health issues and 92% had substance use treatment needs (80). While women account for 7% of the federal and 16% of the provincial custodial population, their needs are overlooked due to the larger population of incarcerated men (75).

Current research does not consider women’s gender-specific health care needs (77, 81, 82), such as access to contraception, abortion, antenatal care and infant care (71–74). Ontario data quantify these gaps: among pregnancies involving time in provincial custody, first-trimester physician visits (30.8%) and first-trimester ultrasonography (34.6%) were uncommon, fewer than half (48.4%) reached the WHO-recommended minimum of eight antenatal contacts, and the odds of a first-trimester visit were roughly one-tenth those of the general population (74). Breastfeeding services, maternity service availability, and geographic isolation of reproductive and women’s-specific clinics further limit essential gender-specific services (75, 83).

Inconsistencies in both policy and healthcare access limit women’s access to support within prison and after release. Evidence across various sources highlighted health care fragmentation driven by short sentences, frequent transfers, and poor coordination with community services (76, 81). Research indicated women were disproportionately affected by HIV, STBBIs, mental illness, and substance use (77). Safety and access concerns for transgender people in correctional centers further underscored the need for inclusive, gender-responsive correctional health services (84).

### Mortality

Premature and preventable mortality emerged as a recurring concern across the reviewed literature (15, 85–88). Drug poisoning (overdose) was identified as a leading cause of preventable death among incarcerated people, particularly in the immediate post-release period, with the highest risk of overdose death occurring within the first 30 days following release (15, 86–88). Indigenous Peoples and Black people experienced an elevated risk of overdose death (89, 90). At the system level, the 2017 transfer of correctional health services to the Ministry of Health in British Columbia was associated with a reduction in deaths following release (85).

## Discussion

This narrative review synthesizes contemporary research on the health of incarcerated adults in Canada. Two findings emerged from the review: (1) People who are incarcerated in Canada experience a disproportionately high burden of disease compared to community-based populations, and (2) Health care access and continuity are often fragmented, particularly during times of transition when mortality risk is high and system responsibility for the delivery of care is low. Because most incarcerated people in Canada return to the community, these health inequities have direct implications for population health. As a result, there is a critical need to expand the scope and depth of research on incarceration and health care in Canada to inform effective policy, improve continuity of care across custodial and community settings, and address structural drivers of health inequities.

In comparison with previous Canadian reviews of the health of incarcerated people, this review captures several developments that the earlier literature could not (14). The COVID-19 pandemic produced outbreak intensity in correctional centers that exceeded that of any other congregate setting in Canada, alongside sustained disruption to routine and chronic disease care. Post-release overdose mortality has since emerged as the most consistently measured harm associated with incarceration in Canada. Population-level data linkage in Ontario and British Columbia has made these harms quantifiable for the first time, while exposing the near-total absence of evidence from the territories and much of Central Canada. Governance reform, notably the transfer of correctional health services to provincial ministries of health, has also become an evaluable intervention rather than a policy proposal.

Incarceration is consistently identified in the literature as both a structural determinant of health and an outcome of cumulative disadvantage in the community. Factors like poverty, racism, barriers to health care, colonialism, and housing insecurity in Canadian communities directly shape who is criminalized and incarcerated, and strongly influence health outcomes. The overincarceration of Indigenous Peoples is a clear example of how structural determinants of health drive criminalization, incarceration, and contemporary population health inequities. The overincarceration of Indigenous Peoples has been recognized as a public health crisis in Canada that requires systems-level interventions that improve care within correctional centers and address conditions in the community (91). The same structural determinants that contribute to criminalization and incarceration persist after release, sustaining a regular cycle of transition in and out of correctional centers that increases the risk of premature mortality (92). Addressing this cycle requires upstream, community-based interventions that target structural inequities alongside culturally safe health and social services in correctional centers that are informed by the knowledge of people with lived experience of incarceration. Distinguishing these pathways is important: some of the disparities described in this review reflect community-level determinants that drive both poor health and the risk of incarceration, whereas others reflect the effects of the carceral system itself, such as disrupted continuity of care following release. Addressing health inequities among people who experience incarceration therefore requires action on both community-level determinants and the conditions of custody.

Research consistently reported that incarceration worsened health. Research described how segregation or seclusion units contribute to mental health decline and exacerbate existing psychiatric symptoms, increase the risk of self-harm, and create significant barriers to accessing health care services (59, 93). The negative impacts of incarceration are not distributed equally among incarcerated people. Indigenous Peoples who are incarcerated are more likely to receive higher security classifications within Canadian correctional settings, and reports of anti-Indigenous racism in Canadian correctional centers are well documented (78, 80, 91). Beyond mental health, the effects of incarceration on health were also evident in the disruption of treatment continuity at transfer and release, which, as noted above, substantially reduced the likelihood of completing treatment for conditions such as hepatitis C.

The geographical distribution of the included studies reveals significant regional gaps in the contemporary literature, particularly in Quebec, the Prairie provinces, Atlantic Canada, and the territories. Results show that research was heavily concentrated in Ontario (*n* = 42) and British Columbia (*n* = 16). The remaining studies were distributed across Alberta, Saskatchewan, and Manitoba (*n* = 13), Quebec (*n* = 4), Nova Scotia (*n* = 2), Newfoundland and Labrador (*n* = 2), and New Brunswick (*n* = 1). No research was identified from Prince Edward Island or the Canadian territories. The lack of evidence from these regions illustrates how the health status and care experiences of many incarcerated people remain largely unknown to the public health community. Given the delivery of health services across 13 provincial and territorial jurisdictions, these gaps hinder the ability to conduct pan-Canadian analyses to identify jurisdictional variations in care and ensure compliance with international standards such as the UN Mandela Rules. This distribution reflects where research has been conducted rather than where correctional facilities are located. Correctional facilities operate in every province and territory, spanning both the federal system and provincial and territorial correctional centers, and the concentration of research in Ontario and British Columbia only partly mirrors their larger incarcerated populations (94). It also reflects established provincial data-linkage infrastructure in these two provinces, which has enabled much of the population-level correctional health research conducted in Canada to date. The absence of research from the territories is especially notable: Yukon, the Northwest Territories, and Nunavut each operate their own correctional facilities and report the highest adult incarceration rates in the country, driven substantially by the overrepresentation of Indigenous Peoples (95). The near-total absence of evidence from these jurisdictions therefore represents one of the most consequential gaps in the current literature rather than a reflection of where incarcerated populations reside.

Furthermore, a notable portion of the literature did not disclose a specific geographical location (*n* = 20). While this omission may be intended to protect the anonymity of specific facilities, it ultimately limits the transparency of the research. Without geographic specificity, it is difficult to pinpoint where the most significant gaps in service delivery exist, further complicating efforts to address fragmented and inconsistent access to healthcare across the Canadian carceral system.

Research highlights that correctional centers are important settings for health care delivery and efforts to improve the social determinants of health. Numerous studies focused on women’s health, STBBIs, substance use disorder, chronic disease, nutrition and aging underscored the importance of intervening while people are incarcerated to improve treatment uptake. Evidence suggests that while some pharmacological treatments are widely available in the community for the pervasive health issues that affect incarcerated people, access is fragmented and inconsistent across correctional jurisdictions in Canada. For example, research emphasized that while OAT was a core intervention for reducing overdose death among incarcerated people, access to OAT and medication access varies greatly across correctional centers (65, 66, 96). The UN Mandela Rules outlines the need for correctional health care services to be equivalent to those in the community. Pan-Canadian research is urgently needed to understand jurisdictional variations in access to pharmaceutical medications and treatments to assess national compliance with international standards for prison health care.

The organizational structure of health service delivery is a critical determinant of whether incarcerated people receive care equivalent to community standards (8). The UN Mandela Rules and WHO advocate for the independence of healthcare, recommending that services be delivered by public health agencies rather than the same organizations responsible for security (8). This separation is intended to ensure that independent clinical decision-making is prioritized over security interests and that correctional health care is integrated with community health systems (97, 98).

In Canada, correctional health governance is highly fragmented across 13 provincial and territorial jurisdictions and the federal government. While 76% of all incarcerated people are held in provincial or territorial centers (94), only British Columbia, Alberta, Nova Scotia, and Newfoundland and Labrador have aligned with international recommendations by transferring healthcare responsibility to their respective ministries of health. In all other jurisdictions, including federal correctional centers, healthcare remains under the same government department as security. Health care governance has direct implications for the delivery of health care in correctional centers. Ministries of Health have the institutional policies and procedures to implement community standards of care, and shifting responsibility to the Ministry of Health can facilitate improved continuity of care and better health outcomes (85). To address existing health inequities, further evaluation of these governance models in Canada is urgently needed (10). Comparing jurisdictions that have transferred health care to ministries of health with those that have not offers a natural opportunity to evaluate whether governance reform improves continuity of care and reduces preventable mortality; such comparative, policy-focused evaluation should be a priority for aligning Canadian correctional health care with the equivalence-of-care principle set out in the UN Mandela Rules.

Other themes that emerged included the declining health status of incarcerated people due to COVID-19 pandemic and the ongoing overdose crisis, which is a major contributor to mortality in the days and weeks after people return to community. These findings reflect the compounding structural factors that impact the health of incarcerated people in Canada, and sedimentation of disease among incarcerated people who experience disproportionately high rates of morbidity and mortality (7).

This review has several limitations. As a narrative rather than systematic review, it did not apply the full PRISMA checklist or a formal quality appraisal of the included studies, and the findings should be interpreted accordingly. The search was restricted to English-language, peer-reviewed literature and excluded grey literature, which may have omitted relevant information. Finally, the available evidence was regionally concentrated in Ontario and British Columbia, with no research identified from the territories, which limits the generalizability of the findings across all Canadian jurisdictions.

## Conclusion

Incarcerated people in Canada experience disproportionately high rates of disease and premature mortality. Incarceration is an important structural determinant of health that intersects with community-based social determinants of health, reproducing health inequities in Canada. To reduce preventable death among people who experience incarceration, efforts are required to strengthen continuity of care, address the social determinants of health, and reverse longstanding structural inequities that contribute to premature mortality among incarcerated people. Research and evaluation are urgently needed to understand disparate approaches to the delivery of health care in correctional centers across Canada.

## Data Availability

The data analyzed in this study is subject to the following licenses/restrictions: data available by request. Requests to access these datasets should be directed to Amanda Slaunwhite, amanda.slaunwhite@ubc.ca.
