# Supplementary material for: The health status of incarcerated people in Canada: a narrative review
Source: Front Public Health. 2026 Sep 11;14:1902956. doi: 10.3389/fpubh.2026.1902956 (PMC13612283; doi:10.3389/fpubh.2026.1902956)
Supplement: Supplementary file 1 [file Supplementary_File_1.docx]

**Supplement A.** Search Strategy

| Database(s) | Search terms | |
| --- | --- | --- |
| Medline | 1 | (Convict* or Correctional* or Felon* or Incarcerat* or Inmate* or Inprison* or Jail* or Offender* or penitentiar* or Prison* or carceral*).mp. [mp=title, book title, abstract, original title, name of substance word, subject heading word, floating sub-heading word, keyword heading word, organism supplementary concept word, protocol supplementary concept word, rare disease supplementary concept word, unique identifier, synonyms, population supplementary concept word, anatomy supplementary concept word] |
|  | 2 | Prisoners/ or Health Services for Prisoners/ |
|  | 3 | incarceration/ or prisons/ |
|  | 4 | 1 or 2 or 3 |
|  | 5 | canada/ or alberta/ or british columbia/ or manitoba/ or new brunswick/ or "newfoundland and labrador"/ or northwest territories/ or nova scotia/ or nunavut/ or ontario/ or prince edward island/ or quebec/ or saskatchewan/ or yukon territory/ |
|  | 6 | 4 and 5 |
|  | 7 | limit 6 to (english language and yr=”2014-Current”) |

| Database | Search terms |
| --- | --- |
| PsycINFO | XB ( carceral or convict* or Correctional* or Criminal* or Felon*or Incarcerat* or Inmate* or Inprison* or Jail* or penitentiar* or prison* ) AND XB ( "Health in prison" or health* or "Public Health" or "prison health" or "Correctional health*" ) AND XB ( Alberta or "British Columbia" or BC or Canada or Canadian* or Labrador or Manitoba or New Brunswick or Newfoundland or "nova scotia" or Nunavut or nwt or "Northwest Territories" or Ontario or "Prince Edward Island" or Quebec or Saskatchewan or Yukon ) |

| Database | Search terms |
| --- | --- |
| Web of Science | TS=( (incarcerat* OR imprison* OR prison* OR jail* OR inmate* OR offender* OR "correctional*" OR "penal*" OR carceral) AND (health OR "public health" OR "mental health" OR "physical health" OR "primary care" OR "health care" OR "health services" OR "health outcomes" OR "reproductive health" OR addiction OR substance* ) ) AND TS=( Canada OR Canadian* OR "British Columbia" OR Alberta OR Saskatchewan OR Manitoba OR Ontario OR Quebec OR "Nova Scotia" OR Newfoundland OR Labrador OR Yukon OR Nunavut OR "Northwest Territories" OR "Prince Edward Island" ) |

| Database | Search terms |
| --- | --- |
| Scopus | ( TITLE-ABS-KEY ( carceral OR convict* OR correctional* OR criminal* OR felon*or AND incarcerat* OR inmate* OR inprison* OR jail* OR penitentiar* OR prison* ) AND TITLE-ABS-KEY ( alberta OR "British Columbia" OR bc OR canada OR canadian* OR labrador OR manitoba OR new AND brunswick OR newfoundland OR "nova scotia" OR nunavut OR nwt OR "Northwest Territories" OR ontario OR "Prince Edward Island" OR quebec OR saskatchewan OR yukon ) ) AND PUBYEAR > 2013 AND PUBYEAR < 2027 AND ( LIMIT-TO ( LANGUAGE , "English" ) ) |

| Database | Search Terms |
| --- | --- |
| CINHAL | XB ( carceral OR convict* OR correctional* OR criminal* OR felon*or AND incarcerat* OR inmate* OR inprison* OR jail* OR penitentiar* OR prison* ) AND XB ( alberta OR "British Columbia" OR bc OR canada OR canadian* OR labrador OR manitoba OR new AND brunswick OR newfoundland OR "nova scotia" OR nunavut OR nwt OR "Northwest Territories" OR ontario OR "Prince Edward Island" OR quebec OR saskatchewan OR yukon ) AND XB ( "Health in prison" or health* or "Public Health" or "prison health" or "Correctional health*" ) Publication Date: 20140101-20260722 |
