# Supplementary material for: The health status of incarcerated people in Canada: a narrative review
Source: Front Public Health. 2026 Sep 11;14:1902956. doi: 10.3389/fpubh.2026.1902956 (PMC13612283; doi:10.3389/fpubh.2026.1902956)
Supplement: Supplementary file 2 [file Table_1.docx]

**Supplementary Table 1. PICOT-style summary of the eligibility criteria**

The Health Status of Incarcerated People in Canada: A Narrative Review

Eligibility criteria applied at both title/abstract and full-text screening, presented in PICOT format (Population, Intervention or exposure, Comparison, Outcome, Time), together with the additional operational criteria used for setting, study design and publication type, and language. Since this narrative review focuses on health status rather than a review of a specific intervention incarceration in a Canadian correctional centre was treated as the exposure of interest and no comparator was required for eligibility. Screening at both stages was completed independently by two reviewers, Disagreements were resolved through discussion, and where consensus could not be reached, by consultation with the senior author. The full reproducible search strategies for all five databases are provided in Supplement A.

Abbreviations: PICOT = Population, Intervention (exposure), Comparison, Outcome, Time.

| **Element** | **Inclusion criteria** | **Exclusion criteria** |
| --- | --- | --- |
| **Population (P)** | Adults aged 18 years or older who were incarcerated in any Canadian correctional setting, including provincial, territorial, and federal correctional centres. Studies in which formerly incarcerated adults reflected on their health during a period of incarceration were also eligible. | People under 18 years of age; correctional staff; people held in forensic mental health facilities; non-human subjects. |
| **Intervention or exposure (I)** | Incarceration in a Canadian correctional centre as the exposure or context of interest, including health status, health conditions, and the health care, screening, treatment, and programming received while in custody. No specific intervention was required for eligibility. | Studies focused exclusively on recidivism, on post-release outcomes, or on economic analyses. |
| **Comparison (C)** | No comparator was required. Studies with and without a comparison group were eligible. Where a comparator was reported (for example, the general or community population, another jurisdiction or facility type, or another time period), it was recorded at data extraction. | Not applicable. The absence of a comparison group was not grounds for exclusion. |
| **Outcome (O)** | Any health-related outcome, finding, or experience of incarcerated adults, spanning the domains synthesized in this review: physical health, mental health, substance use, women's health, sexual and reproductive health, Indigenous Peoples' health, health care utilization, and social determinants of health. | Publications reporting only criminological, legal, or economic outcomes (for example, reoffending, sentencing, or cost analyses) with no health-related outcome. |
| **Time (T)** | Peer-reviewed literature published between January 1, 2014 and July 22, 2026. The start date was selected to build on the previous review by Kouyoumdjian et al., the search for which was completed in 2014. Findings had to pertain to health during a period of incarceration. | Publications dated before January 1, 2014. Studies addressing only the period following release from custody. |
| **Additional operational criteria** | | |
| **Setting** | Canada, including provincial, territorial, and federal correctional centres and both remand and sentenced custody. | Research conducted outside Canada; research conducted in forensic mental health facilities. |
| **Study design and publication type** | Peer-reviewed publications of any design, including original research (quantitative, qualitative, and mixed methods), reviews, commentaries, and reports. | Grey literature, including dissertations and other non-peer-reviewed sources. Grey literature was excluded to focus the synthesis on peer-reviewed evidence and to support the reliability and reproducibility of the search process. |
| **Language** | Published in English. | Published in a language other than English. |
