# Supplementary material for: The health status of incarcerated people in Canada: a narrative review
Source: Front Public Health. 2026 Sep 11;14:1902956. doi: 10.3389/fpubh.2026.1902956 (PMC13612283; doi:10.3389/fpubh.2026.1902956)
Supplement: Supplementary file 3 [file Table_2.docx]

**Supplementary Table 2. Characteristics of included studies**

The Health Status of Incarcerated People in Canada: A Narrative Review

Characteristics of the 167 articles included in the review, listed alphabetically by first author. Article type follows the classification applied during data extraction (original research, review, commentary, report, other); the study design shown after the em dash is the design reported by the study authors. Health domain reflects the primary and secondary literature classifications assigned during data extraction; many studies address more than one domain, so domain counts exceed the number of studies. Incarceration status indicates whether participants were in custody when the data were collected; where the data extraction form recorded no value, this is shown as not reported. Period relative to COVID-19 was classified as pre-COVID-19, during COVID-19, or post-COVID-19 (data collected after 2023).

Population and setting is presented as [target population] in a [jurisdiction] correctional centre in [province], where these were reported by the source study. [Jurisdiction] = provincial/territorial or federal.

Abbreviations: BMI = body mass index; CSC = Correctional Service Canada; DUNDRUM = Dangerousness, Understanding, Recovery and Urgency Manual; HCV = hepatitis C virus; HIV = human immunodeficiency virus; interRAI = international Resident Assessment Instrument; Pap = Papanicolaou; RNA = ribonucleic acid; STBBI = sexually transmitted and blood-borne infection; STI = sexually transmitted infection.

| **No.** | **Study (first author, year, journal)** | **Article type and Research Design** | **Population and setting** | **Health domain(s)** | **Outcome(s) or focus of measurement** | **Incarceration status at time of study; period relative to COVID-19** |
| --- | --- | --- | --- | --- | --- | --- |
| 1 | Ahmed R, et al. (2016), Int J Prison Health | Original research — qualitative descriptive | Women in a provincial/territorial remand centre in Alberta | Women's health; health care use | Barriers to accessing health care in custody; continuity of care at transfer and release | Incarcerated; pre-COVID-19 |
| 2 | Ahmed RA, et al. (2016), J Correct Health Care | Original research — qualitative descriptive | Women in a provincial/territorial remand centre in Alberta | Women's health; social determinants of health | Homelessness, health service requests, cancer screening, transition to community | Incarcerated; pre-COVID-19 |
| 3 | Albaini O, et al. (2025), Can Liver J | Original research — national cross-sectional survey (88 of 94 prisons) | People in provincial/territorial prisons across Canada, 2020–2024 | Physical health; public health and infection control; treatment | Change in HCV screening approach, reflex RNA testing, direct-acting antiviral provision and linkage to care at release | Other; post-COVID-19 |
| 4 | Bancroft X, et al. (2025), BMC Pregnancy Childbirth | Original research — qualitative descriptive (n=8) | People incarcerated while pregnant in federal and provincial/territorial custody in Ontario, interviewed after release | Sexual and reproductive health; women's health; health care use | Access to perinatal care in custody and after release; carceral constraints on health and social supports, mistreatment and parenting | Not incarcerated; post-COVID-19 |
| 5 | Bartlett S, et al. (2021), Clin Liver Dis | Review | People in federal and provincial/territorial custody across Canada | Physical health | HCV prevalence, screening coverage and direct-acting antiviral treatment access | Incarcerated; during COVID-19 |
| 6 | Beaudette JN, Stewart LA (2016), Can J Psychiatry | Original research — national cross-sectional survey | Men entering federal custody across Canada | Mental health; substance use | Prevalence of current mental disorders and comorbidity with antisocial personality and substance use disorder | Incarcerated; pre-COVID-19 |
| 7 | Besney J, et al. (2017), J Infect Prev | Report — outbreak investigation | People incarcerated and staff during an influenza outbreak in a provincial/territorial correctional centre in Alberta | Public health and infection control | Outbreak control measures, transmission, and barriers to outbreak management in custody | Incarcerated; pre-COVID-19 |
| 8 | Besney JD, et al. (2018), J Correct Health Care | Original research — cross-sectional | 109 women in a provincial/territorial remand centre in Alberta | Women's health; mental health | Mental illness, intimate partner violence, substance use, STI positivity, Pap testing | Incarcerated; pre-COVID-19 |
| 9 | Bhat A, et al. (2020), J Correct Health Care | Report — service evaluation | 1,500 people admitted to a provincial/territorial remand centre in Alberta (Edmonton Remand Centre) | Health care use | Proportion assessed by a pharmacist within 48 hours of admission; type of intervention | Incarcerated; during COVID-19 |
| 10 | Bodkin C, et al. (2021), BMJ Open | Original research — repeated cross-sectional | People in provincial/territorial prisons in Ontario, 2015–2018 | Treatment; substance use | Rates of opioid agonist treatment prescribing over time versus the community | Other; during COVID-19 |
| 11 | Boyer Y, et al. (2019), Can J Women Law | Report — legal and policy analysis | Trans people in federal custody across Canada | Sexual and reproductive health | Sexual violence and safety; access to gender-affirming hormone therapy | Incarcerated; pre-COVID-19 |
| 12 | Bozinoff N, et al. (2018), Drug Alcohol Depend | Original research — prospective cohort | People with opioid use disorder and recent federal or provincial/territorial incarceration in British Columbia (Vancouver) | Treatment | Opioid agonist treatment utilization in custody; non-fatal overdose and continuity after release | Incarcerated; pre-COVID-19 |
| 13 | Brink J (2018), Can J Psychiatry | Commentary | People in federal custody across Canada prescribed psychotropic medication | Mental health | Off-label psychotropic prescribing; self-injurious and suicidal behaviour | Incarcerated; pre-COVID-19 |
| 14 | Brown GP, et al. (2015), Int J Offender Ther Comp Criminol | Original research — cross-sectional (interRAI) | Men in provincial/territorial correctional facilities in Ontario | Mental health | Prevalence of current severe mental health symptoms versus a community sample | Incarcerated; pre-COVID-19 |
| 15 | Brown GP, et al. (2018), Can J Psychiatry | Original research — cross-sectional | Federally sentenced men and women across Canada | Treatment | Rates of approved versus off-label psychotropic prescribing by sex and age | Incarcerated; pre-COVID-19 |
| 16 | Brown A, et al. (2019), Univ Chicago Press J | Report — program evaluation | Mothers in federal custody across Canada, their children and caregivers | Women's health | Parent–child attachment, parenting capacity, maternal anxiety and depression | Incarcerated; pre-COVID-19 |
| 17 | Brown C, et al. (2026), J Obstet Gynaecol Can | Original research — policy review (49 responding hospitals) | Maternity hospitals providing care to people in federal and provincial/territorial custody across Canada | Sexual and reproductive health; women's health; health care use | Existence and content of maternity hospital policies; restraint use and staff roles during labour and birth | Other; post-COVID-19 |
| 18 | Bucerius SM, Haggerty KD (2019), Int J Drug Policy | Original research — qualitative | People incarcerated and correctional officers in provincial/territorial facilities in Western Canada | Substance use | Fentanyl availability, overdose risk and fear of inadvertent contamination | Incarcerated; pre-COVID-19 |
| 19 | Bucerius S, et al. (2020), Br J Criminol | Original research — qualitative (n=88) | Women in provincial/territorial remand facilities in Western Canada | Women's health; social determinants of health | Prison as temporary refuge: victimization, housing, food, health access, programming | Incarcerated; during COVID-19 |
| 20 | Bucerius S, Sandberg S (2022), Crime Justice | Review | Women in prison in Canada, Mexico and Norway | Women's health | Lifetime victimization, drug use, gendered work and motherhood, access to basic necessities | Incarcerated; during COVID-19 |
| 21 | Burki TK (2017), Lancet Oncol | Commentary | People in correctional facilities across Canada | Physical health | Cancer incidence by site versus the general population | Incarcerated; pre-COVID-19 |
| 22 | Butler A, et al. (2021), Psychiatr Serv | Original research — repeated cross-sectional (linked administrative data) | People admitted to provincial/territorial prisons in British Columbia over 9 years | Mental health; substance use | Prevalence of mental health need, substance use disorder and co-occurring disorders | Incarcerated; during COVID-19 |
| 23 | Butler A, et al. (2023), PLoS One | Original research — whole-population data linkage | People who experienced provincial/territorial incarceration in Ontario, 2015–2020, vs the general population | Physical health; mortality | Opioid toxicity death before and during the COVID-19 pandemic, by sex and time since release | Incarcerated; during COVID-19 |
| 24 | Butsang T, et al. (2025), Lancet Reg Health Am | Original research — whole-population retrospective cohort | Indigenous people who experienced provincial/territorial incarceration in Ontario, 2015–2020 | Mortality | Opioid toxicity mortality rates versus people not incarcerated, by sex | Incarcerated; post-COVID-19 |
| 25 | Button L, et al. (2023), Forensic Sci Med Pathol | Original research — retrospective review of coroner data | People who died in federal and provincial/territorial correctional facilities in Ontario, 2009–2019 | Mortality | Opioid-related deaths over time; substances detected; manner of death | Incarcerated; during COVID-19 |
| 26 | Cameron C, et al. (2021), BJPsych Int | Report | People with mental disorder in federal custody across Canada | Mental health | Mental health service delivery, segregation, consent, violence and suicide risk | Incarcerated; during COVID-19 |
| 27 | Carter-Ramirez A, et al. (2020), JAMA Netw Open | Original research — retrospective cohort (linked administrative data) | Pregnant people who experienced provincial/territorial imprisonment in Ontario vs the general population | Women's health | Antenatal care quality indicators (first-trimester visit, visit adequacy, screening) | Incarcerated; during COVID-19 |
| 28 | Carter-Ramirez A, et al. (2020), J Obstet Gynaecol Can | Original research — retrospective cohort (linked administrative data) | Pregnant people who experienced provincial/territorial imprisonment in Ontario vs the general population | Women's health | Infant and maternal outcomes; parity, HIV, viral hepatitis and mental health diagnoses | Incarcerated; during COVID-19 |
| 29 | Cervin C (2021), Can Fam Physician | Commentary | People in prisons across Canada | Public health and infection control | COVID-19 vaccine prioritization and equity in congregate carceral settings | Incarcerated; during COVID-19 |
| 30 | Christensen LCJ, Egli SL (2026), Can J Emerg Nurs | Report — practice reflection on two mass evacuations | People evacuated to a large provincial/territorial correctional centre in Alberta following wildfires, 2023–2024 | Health care use; public health and infection control | Disaster preparedness for mass admission: client movement, transfer of health information, supplies and staffing | Incarcerated; post-COVID-19 |
| 31 | Courtemanche Y, et al. (2018), Can J Public Health | Original research — repeat cross-sectional (2003 vs 2014–15) | Adults in provincial/territorial detention centres in Quebec | Public health and infection control; physical health | HIV and HCV prevalence; injection, tattooing and sexual risk behaviours | Incarcerated; pre-COVID-19 |
| 32 | Craig DF (2015), Can J Addict | Original research — practice evaluation | Adults in a provincial/territorial prison in Newfoundland and Labrador prescribed psychotropic medication | Mental health | Psychotropic deprescribing; reported prison violence and mental health | Incarcerated; pre-COVID-19 |
| 33 | Cuthbertson L, et al. (2018), J Correct Health Care | Original research — mixed methods | People in a provincial/territorial remand centre in Alberta | Treatment | Patient-, provider- and system-level factors affecting medication adherence | Incarcerated; pre-COVID-19 |
| 34 | Dam D, et al. (2024), BMC Public Health | Original research — retrospective analysis of outbreak surveillance data | Congregate settings across Canada, including correctional facilities | Public health and infection control | COVID-19 outbreak intensity, size and duration by setting type | Not reported; post-COVID-19 |
| 35 | De Graaf K, Kilty JM (2016), Punishm Soc | Original research — qualitative | Women in federal and provincial/territorial custody in Ontario | Physical health; women's health | Prison food quality, nutrition, gastrointestinal symptoms and weight change | Incarcerated; pre-COVID-19 |
| 36 | Dell C, et al. (2019), Int J Prison Health | Original research — modified instrumental case study (n=3) | Men in a federal psychiatric prison in Saskatchewan | Mental health; treatment | Human–animal bond in canine animal-assisted therapy; recognition of emotions and institutional conduct | Incarcerated; pre-COVID-19 |
| 37 | Erickson M, et al. (2022), Can J Public Health | Original research — qualitative | Women living with HIV and experience of provincial/territorial incarceration in British Columbia | Physical health; women's health | HIV-related stigma, confidentiality and access to HIV care in custody | Incarcerated; during COVID-19 |
| 38 | Evans J, Mussell L (2023), Can J Law Soc | Original research — qualitative interviews and discourse analysis | People in provincial/territorial prisons and jails in Ontario during the COVID-19 pandemic | Mental health; physical health; social determinants of health | Harms of forced confinement and isolation; legitimation of segregation-like practices during the pandemic | Incarcerated; during COVID-19 |
| 39 | Farrell-MacDonald S, et al. (2022), Int J Drug Policy | Original research — retrospective cohort | Men with opioid dependence in federal custody across Canada | Treatment | Retention on methadone versus buprenorphine/naloxone; post-release treatment and recidivism | Incarcerated; during COVID-19 |
| 40 | Fayter R, et al. (2021), Can J Sociol | Original research — qualitative analysis of lived-experience accounts | People in federal prisons across Canada during the COVID-19 pandemic | Mental health | Mental health impacts of pandemic crisis governance; isolation, loss of programming and suspended visits | Incarcerated; during COVID-19 |
| 41 | Filoso D, et al. (2026), Int J Prison Health | Original research — cross-sectional survey (CSC National Health Survey, n=413) | People in federal prisons across Canada, 2022 | Substance use; public health and infection control | Substance use frequency and route; sharing of snorting and injecting equipment | Incarcerated; during COVID-19 |
| 42 | Fisher M, et al. (2026), Can J Nurs Res | Original research — qualitative | Nurses providing cancer care to people who experience provincial/territorial incarceration in British Columbia | Physical health; health care use; treatment | Barriers to cancer care delivery: security priorities, stigma, confidentiality, continuity and dual loyalty | Other; post-COVID-19 |
| 43 | Flannigan K, et al. (2025), Int J Law Psychiatry | Review — updated systematic review | People with fetal alcohol spectrum disorder in the criminal legal system | Physical health | Incarceration, offending and victimization by age, sex and Indigenous identity | Incarcerated; post-COVID-19 |
| 44 | Fuller C, et al. (2026), Psychol Public Policy Law | Original research — qualitative (n=9) | Transgender and gender-diverse people with experience of federal custody across Canada | Health care use; mental health; social determinants of health | Access to gender-affirming and general health care; placement, segregation and surveillance under Commissioner's Directive 100; reintegration | Not incarcerated; post-COVID-19 |
| 45 | Gacek J, Molloy E (2026), Mortality | Original research — qualitative analysis of natural deaths in custody | Older people who died of natural causes in federal custody across Canada | Mortality; health care use | Palliative and end-of-life care in custody; compassionate release and oversight of quality-of-care review | Other; post-COVID-19 |
| 46 | Gratix J, et al. (2019), Int J Prison Health | Review [article type as coded; reported as a cross-sectional evaluation] | Admissions to three provincial/territorial correctional facilities in Alberta | Physical health | Opt-in STBBI screening uptake and positivity (chlamydia, syphilis, HIV, HCV) | Incarcerated; pre-COVID-19 |
| 47 | Harper K, et al. (2018), Can J Surg | Original research — retrospective cohort | 20 men in federal custody in Ontario undergoing 25 hip or knee arthroplasties over 10 years | Physical health | Major surgical complications, infection and revision surgery versus a comparison group | Incarcerated; pre-COVID-19 |
| 48 | Heggie C, et al. (2026), PLoS One | Original research — cross-sectional survey (n=75; 48% Indigenous) | People in a provincial/territorial prison for women in British Columbia | Sexual and reproductive health; women's health | Pregnancy and abortion history, contraceptive use, Pap testing and mammography | Incarcerated; post-COVID-19 |
| 49 | Hopley A, Brunelle C (2016), Addict Behav | Original research — cross-sectional | Incarcerated men in federal and provincial/territorial custody in New Brunswick | Substance use | Personality profile (sensation seeking, anxiety sensitivity) predicting institutional substance use | Incarcerated; pre-COVID-19 |
| 50 | Hyatt A (2019), First Peoples Child Fam Rev | Commentary | Indigenous people in correctional facilities across Canada | Indigenous health | Cultural healing and rehabilitation; residential school legacy, education and employment barriers | Incarcerated; pre-COVID-19 |
| 51 | Iftene A (2016), Int J Law Psychiatry | Original research — cross-sectional interviews (n=197) | Men aged 50 and older in federal penitentiaries across Canada | Mental health | Self-reported mental illness, psychiatric follow-up, suicidal ideation and use of segregation | Incarcerated; pre-COVID-19 |
| 52 | Iftene A (2017), Can J Criminol Crim Justice | Original research — cross-sectional interviews (n=197) | Men aged 50 and older in federal penitentiaries across Canada | Physical health | Self-rated health, chronic disease burden and chronic pain management in an ageing population | Incarcerated; pre-COVID-19 |
| 53 | Iftene A (2020), Can Fam Physician | Commentary | Older adults in federal penitentiaries across Canada | Physical health | Comorbidity, dementia, terminal illness, access to pain medication and COVID-19 infection rates | Incarcerated; during COVID-19 |
| 54 | Johansson J, et al. (2023), J Forensic Nurs | Report | Ageing people convicted of sexual offences in federal custody across Canada | Physical health | Accelerated ageing, end-of-life needs and access to compassionate release | Incarcerated; during COVID-19 |
| 55 | Johnson C, et al. (2018), CMAJ Open | Original research — retrospective cohort | Adults in federal penitentiaries across Canada | Physical health | Weight change during incarceration | Incarcerated; during COVID-19 |
| 56 | Johnson C, et al. (2018), Can J Public Health | Original research — retrospective cohort | Adults in federal penitentiaries across Canada | Physical health | Influence of physical activity, screen time and sleep on body weight and BMI | Incarcerated; pre-COVID-19 |
| 57 | Johnson C, et al. (2018), PLoS One | Original research — retrospective cohort | Adults in federal penitentiaries across Canada who gained weight in custody | Physical health | Reported food intake, canteen purchasing and weight gain | Incarcerated; pre-COVID-19 |
| 58 | Johnson C, et al. (2019), BMJ Open | Original research — cohort study | Adults in federal penitentiaries across Canada, by tobacco use history | Physical health | Weight and obesity change following the federal tobacco ban | Incarcerated; pre-COVID-19 |
| 59 | Johnson C, et al. (2021), Int J Prison Health | Original research — retrospective cohort | Adults in federal penitentiaries across Canada | Physical health | Weight gain and progression of obesity-related chronic disease during incarceration | Incarcerated; during COVID-19 |
| 60 | Johnson C, et al. (2021), J Correct Health Care | Original research — retrospective cohort | Adults in federal penitentiaries across Canada | Physical health | Association of weight gain with mental health diagnoses and psychotropic medication use | Incarcerated; during COVID-19 |
| 61 | Johnson C, et al. (2022), Nutrients | Original research — nutritional analysis | National menu served in federal penitentiaries across Canada | Physical health | Nutritional adequacy of the national menu, including sodium content | Not incarcerated; during COVID-19 |
| 62 | Johnson C (2023), Can J Diet Pract Res | Original research — methodological reflection | Adults in federal correctional facilities across Canada | Physical health | Feasibility and challenges of conducting nutrition and health research in custody | Incarcerated; during COVID-19 |
| 63 | Johnson C, et al. (2024), Int J Prison Health | Original research — cross-sectional (n=754) | Adults in federal correctional facilities across Canada | Physical health | Association between social support networks and anthropometric change | Incarcerated; post-COVID-19 |
| 64 | Jones DJ, et al. (2019), J Community Saf Well-Being | Original research — qualitative | Women in provincial/territorial remand in Western Canada | Health care use; physical health; substance use; women's health | Victimization histories, addiction and mental health, parenting while incarcerated | Incarcerated; pre-COVID-19 |
| 65 | Jones RM, et al. (2019), BMC Psychiatry | Original research — cross-sectional | All women in provincial/territorial custody in Ontario | Mental health; women's health | Proportion requiring psychiatric hospital admission; DUNDRUM-1/2 psychometric validity | Incarcerated; pre-COVID-19 |
| 66 | Jones R, et al. (2024), Health Justice | Original research — qualitative (focus groups) | Women in a provincial/territorial prison in Ontario | Women's health | Contraception knowledge, decision-making and access during incarceration | Incarcerated; post-COVID-19 |
| 67 | Jones RM, et al. (2026), J Am Acad Psychiatry Law | Original research — retrospective longitudinal observational study (2,288 individuals; 3,273 segregation encounters) | People placed in segregation in provincial/territorial correctional facilities in British Columbia, 2019–2022 | Mental health | Change in Clinical Global Impression–Corrections severity during short-term segregation, by identified mental health need | Incarcerated; during COVID-19 |
| 68 | Jurgutis J, et al. (2025), Soc Sci Med | Review — scoping review (8 articles, 2 Canadian) | Indigenous women in prison in Australia, Canada, New Zealand and the USA | Sexual and reproductive health; Indigenous health; women's health | Reproductive and maternal child health; intergenerational trauma, culturally safe care and family separation | Other; during COVID-19 |
| 69 | Keen C, et al. (2021), Lancet Public Health | Original research — self-controlled case series (linked administrative data) | People who had a non-fatal overdose in British Columbia (provincial/territorial) | Mortality; substance use | Periods of altered non-fatal overdose risk, including prison admission and the weeks after release | Other; during COVID-19 |
| 70 | Kelsall D (2014), CMAJ | Commentary | People held in solitary confinement in prisons across Canada | Mental health | Mental health harms of solitary confinement; self-harm and suicide risk | Not reported; pre-COVID-19 |
| 71 | Knittel A, Sufrin C (2020), Obstet Gynecol | Commentary | Pregnant people who experience incarceration | Sexual and reproductive health; women's health | Quality of antenatal care; monitoring of pregnancy in custody; continuity after release | Incarcerated; during COVID-19 |
| 72 | Kouyoumdjian FG, McIsaac KE (2015), Can J Public Health | Commentary | People in correctional facilities across Canada | Physical health | HCV prevalence and the case for custody as a prevention and control opportunity | Incarcerated; pre-COVID-19 |
| 73 | Kouyoumdjian F, et al. (2016), Can Fam Physician | Review — narrative review | People in federal and provincial/territorial custody across Canada | Mental health; physical health; social determinants; health care use; substance use | Summary of health status: demographics, socioeconomic position, infectious and chronic disease, mental illness | Incarcerated; pre-COVID-19 |
| 74 | Kouyoumdjian FG, et al. (2017), PLoS One | Original research — population-based retrospective cohort | People who experienced provincial/territorial incarceration in Ontario, 2000–2012 | Physical health | Cancer prevalence, incidence and mortality by site and sex | Incarcerated; pre-COVID-19 |
| 75 | Kouyoumdjian FG, et al. (2018), PLoS One | Original research — cross-sectional survey (27 physicians) | Physicians practising in the 26 provincial/territorial correctional facilities for adults in Ontario | Treatment; substance use | Methadone and buprenorphine/naloxone prescribing and initiation in custody; barriers and facilitators | Other; pre-COVID-19 |
| 76 | Kouyoumdjian FG, et al. (2018), PLoS One | Original research — population-based cohort | People released from provincial/territorial prison in Ontario vs a matched general population | Health care use | Ambulatory, emergency and psychiatric health care utilization in prison and after release | Not incarcerated; pre-COVID-19 |
| 77 | Kouyoumdjian FG, et al. (2018), JAMA Netw Open | Original research — retrospective cohort (linked administrative data) | Women admitted to provincial/territorial prison in Ontario vs the general population | Physical health; women's health | Being overdue for cervical cancer screening at index and at 3 years | Other; pre-COVID-19 |
| 78 | Kouyoumdjian FG, et al. (2019), AIDS Care | Original research — retrospective cohort (linked administrative data) | People with HIV who experienced provincial/territorial imprisonment in Ontario | Physical health | HIV care engagement and antiretroviral therapy use in prison and after release | Not incarcerated; during COVID-19 |
| 79 | Kouyoumdjian FG, et al. (2020), PLoS One | Original research — retrospective cohort (linked administrative data) | People who experienced provincial/territorial imprisonment in Ontario vs a matched general population | Physical health | 30-day readmission after medical–surgical hospitalization | Other; during COVID-19 |
| 80 | Kouyoumdjian FG, Orkin AM (2020), Healthc Q | Report | People who experience provincial/territorial imprisonment in Ontario | Health care use | Disease burden, primary care access before and after release, and post-release risk | Incarcerated; during COVID-19 |
| 81 | Krajden M, et al. (2018), Can Liver J | Review | People with HCV in Canada, including correctional populations | Physical health; mortality | HCV epidemiology, prevalence in custody and premature mortality | Incarcerated; pre-COVID-19 |
| 82 | Kronfli N, Cox J (2018), CMAJ | Commentary | People with HCV in provincial/territorial prisons across Canada | Physical health | Barriers to HCV treatment and continuity of care at transfer and release | Incarcerated; pre-COVID-19 |
| 83 | Kronfli N, et al. (2019), Can Liver J | Review | People in correctional facilities across Canada | Physical health; health care use | HCV care cascade: screening, on-site capacity and linkage to care after release | Incarcerated; pre-COVID-19 |
| 84 | Kronfli N, et al. (2021), Can Liver J | Original research — national cross-sectional survey (59 prisons) | Adult provincial/territorial prisons across Canada | Physical health | Variation in HCV screening, treatment and referral practices by governing ministry | Incarcerated; during COVID-19 |
| 85 | Kronfli N, et al. (2022), Clin Infect Dis | Original research — cross-sectional serosurvey | Incarcerated adult men in provincial/territorial custody in Quebec, 2021 | Physical health; public health and infection control | SARS-CoV-2 seroprevalence and associated carceral and social risk factors | Incarcerated; during COVID-19 |
| 86 | Kronfli N, et al. (2024), Front Public Health | Original research — retrospective analysis of laboratory data | Two large provincial/territorial prisons in Montreal, Quebec, July 2018 – February 2022 | Physical health; public health and infection control | Change in HCV antibody and RNA testing before, during and after prison COVID-19 outbreaks | Incarcerated; during COVID-19 |
| 87 | Kurdyak P, et al. (2022), Can J Psychiatry | Original research — retrospective cohort (linked administrative data) | Adults in provincial/territorial jails in Ontario | Health care use; mental health; substance use | Mental health and addiction service use in the 5 years before and during incarceration | Incarcerated; during COVID-19 |
| 88 | Kurz M, et al. (2021), Addiction | Original research — interrupted time series (linked administrative data) | People with opioid use disorder in provincial/territorial correctional facilities in British Columbia | Treatment | Change in opioid agonist treatment uptake following policy change and the overdose emergency | Incarcerated; during COVID-19 |
| 89 | Lachapelle S, Kilty JM (2024), Food Foodways | Original research — qualitative | People in federal prisons across Canada | Physical health | Food quality, portion size, malnutrition and chronic hunger; colonial continuities | Incarcerated; post-COVID-19 |
| 90 | Lafferty L, et al. (2024), Int J Drug Policy | Original research — nominal group technique | People in federal prisons across Canada, peer advocates and staff | Physical health; substance use | Barriers and solutions to prison needle exchange program uptake | Incarcerated; post-COVID-19 |
| 91 | Lawson J (2014), CMAJ | Commentary | Older adults in federal penitentiaries across Canada | Physical health | Chronic disease burden, mobility and infrastructure in an ageing prison population | Incarcerated; pre-COVID-19 |
| 92 | Lessard D, et al. (2022), Vaccine X | Original research — qualitative | People incarcerated in federal prisons across Canada | Public health and infection control | Barriers and facilitators to COVID-19 vaccine acceptability | Incarcerated; during COVID-19 |
| 93 | Leung JW, et al. (2019), J Correct Health Care | Original research — outbreak investigation | People incarcerated and staff in a federal correctional facility in Ontario | Physical health; public health and infection control | Cryptosporidiosis outbreak source, case finding and underreporting of illness | Incarcerated; pre-COVID-19 |
| 94 | Liauw J, et al. (2016), J Obstet Gynaecol Can | Original research — cross-sectional survey | Women in provincial/territorial custody in Ontario | Sexual and reproductive health | Unintended pregnancy, abortion history and unmet contraceptive need | Incarcerated; pre-COVID-19 |
| 95 | Liauw J, et al. (2021), PLoS One | Original research — qualitative | Women in provincial/territorial prisons in Ontario | Sexual and reproductive health; women's health | Experiences of pregnancy and contraceptive care; reproductive safety and dignity | Incarcerated; during COVID-19 |
| 96 | Lunsky Y, et al. (2024), Crim Behav Ment Health | Original research — cross-sectional (linked administrative data) | People in the criminal justice and forensic mental health systems in Ontario (federal and provincial/territorial) | Mental health | Prevalence and clinical profile of intellectual and developmental disabilities | Incarcerated; post-COVID-19 |
| 97 | MacKenzie C, Amirault J (2021), J Correct Health Care | Review | People in institutional settings across Canada and on reintegration | Health care use; mental health | Delivery of institutional mental health care and reintegration supports | Incarcerated; during COVID-19 |
| 98 | Marmel A, Bozinoff N (2020), Int J Prison Health | Report | People with opioid use disorder in prisons across Canada | Treatment; substance use; mortality | Punitive discontinuation of opioid agonist treatment; withdrawal, relapse and overdose risk | Incarcerated; during COVID-19 |
| 99 | Martin MS, et al. (2015), Law Hum Behav | Original research — cohort study | Federally sentenced adults across Canada in their first 180 days of incarceration | Mental health; substance use | Childhood trauma, mental health and substance use as predictors of institutional violence | Incarcerated; pre-COVID-19 |
| 100 | Martin RE, et al. (2016), Glob Health Promot | Original research — community-based participatory feasibility study | Men in a federal correctional facility in British Columbia | Physical health | Feasibility of a peer HIV-prevention intervention; stigma, knowledge and HIV testing uptake | Incarcerated; pre-COVID-19 |
| 101 | Martin MS, et al. (2018), Can J Psychiatry | Original research — cohort study | Federally sentenced adults screened at intake across Canada | Mental health; treatment | Mental health treatment access by sex, race, age and region in relation to screening result | Incarcerated; pre-COVID-19 |
| 102 | Martin MS, et al. (2018), J Consult Clin Psychol | Original research — cohort study | Federally sentenced adults screened at intake across Canada | Mental health; treatment | Patterns of mental health treatment use (brief, acute, chronic) after universal screening | Incarcerated; pre-COVID-19 |
| 103 | Martin MS, et al. (2018), Int J Forensic Ment Health | Original research — propensity score matched cohort | Federally sentenced adults across Canada | Mental health; substance use | Violence, victimization, self-harm, overdose and mortality following mental health screening | Incarcerated; pre-COVID-19 |
| 104 | Matheson FI, et al. (2015), Women Crim Justice | Original research — qualitative | Federally sentenced women with trauma histories across Canada | Mental health; women's health | Need for trauma screening and gender-responsive, trauma-informed treatment | Not incarcerated; pre-COVID-19 |
| 105 | Matheson FI, et al. (2022), J Appl Res Intellect Disabil | Original research — retrospective cohort (linked administrative data) | Adults in a first federal incarceration in Ontario, 2002–2011, vs non-incarcerated groups | Mental health; health care use | Prevalence of intellectual/developmental disabilities; pre-incarceration morbidity, institutional disciplinary charges and post-release health care use | Incarcerated; pre-COVID-19 |
| 106 | McCandless LC, et al. (2015), J Epidemiol Community Health | Original research — cohort with mediation analysis | People with mental illness and criminal justice contact in British Columbia (provincial/territorial) | Mental health; mortality | Mortality and the mediating effect of criminal sentencing rate | Incarcerated; pre-COVID-19 |
| 107 | McConnon A, et al. (2019), Am J Prev Med | Original research — retrospective cohort (linked administrative data) | People released from provincial/territorial correctional facilities in Ontario vs the general population | Physical health | Being overdue for colorectal and breast cancer screening at admission and at 3 years | Incarcerated; pre-COVID-19 |
| 108 | McIntyre JL (2018), J Forensic Nurs | Report — case report | A young man with fentanyl dependence in provincial/territorial custody in Alberta | Substance use; treatment | Barriers to initiating opioid agonist therapy in custody; diversion and recidivism | Incarcerated; pre-COVID-19 |
| 109 | McIsaac KE, et al. (2016), CMAJ Open | Original research — population-based cohort (linked administrative data) | 1.4 million adults aged 18–28 in Ontario, followed 1997–2011 (federal) | Physical health; social determinants of health | Association between traumatic brain injury and subsequent federal incarceration, by sex | Not incarcerated; pre-COVID-19 |
| 110 | McKendy L (2018), Sociol Healthc | Original research — qualitative narrative interviews | Women in a provincial/territorial jail in Ontario | Women's health | Denial of medication and treatment, limited prenatal care and health effects of confinement | Not incarcerated; pre-COVID-19 |
| 111 | McKendy L, et al. (2021), Int J Drug Policy | Original research — retrospective analysis of incident data | People in federal custody across Canada, 2012–2019 | Substance use; mortality | Number, nature, circumstances and outcomes of overdose incidents in custody | Incarcerated; during COVID-19 |
| 112 | McLeod LJ, et al. (2023), J Obstet Gynaecol Can | Original research — retrospective cohort (linked administrative data) | Pregnant people who experienced provincial/territorial incarceration in Ontario vs the general population | Women's health; health care use | Receipt of antenatal obstetrician care and number of antenatal visits | Incarcerated; pre-COVID-19 |
| 113 | McLeod KE, et al. (2024), PLoS One | Original research — retrospective cohort (linked administrative data) | People released from provincial/territorial correctional facilities in British Columbia | Substance use; treatment | Association of opioid agonist treatment in custody with non-fatal overdose in the 30 days after release | Incarcerated; pre-COVID-19 |
| 114 | McLeod KE, et al. (2026), Prim Health Care Res Dev | Original research — qualitative | People with experience of provincial/territorial correctional facilities in Ontario | Health care use | Voicing experiences of health care; gatekeeping, fear of repercussions and patient feedback processes | Not incarcerated; post-COVID-19 |
| 115 | Memarpour P, et al. (2015), Int J Prison Health | Original research — qualitative | Former federal prisoners across Canada | Health care use | Wait times, health care request processes and quality of interactions with providers | Not incarcerated; pre-COVID-19 |
| 116 | Mofina A, et al. (2024), J Correct Health Care | Original research — cross-sectional (interRAI assessment) | Older adults in federal custody across Canada | Mental health; physical health | Prevalence and predictors of pain and depressive symptoms | Incarcerated; post-COVID-19 |
| 117 | Murdoch DJ (2020), Victims Offenders | Original research — policy case study | Provincial/territorial correctional centres in British Columbia during the COVID-19 pandemic | Physical health; public health and infection control | Screening, induction units and isolation practices; prevention of in-custody COVID-19 spread | Incarcerated; during COVID-19 |
| 118 | Nickel NC, et al. (2024), Womens Health Issues | Original research — linked administrative data study | First Nations and other birthing parents incarcerated while pregnant in provincial/territorial custody in Manitoba | Sexual and reproductive health; Indigenous health | Adverse birth outcomes, including low birthweight, by timing of incarceration | Incarcerated; post-COVID-19 |
| 119 | Nolan AM, Stewart LA (2017), J Correct Health Care | Original research — cross-sectional | Women entering federal custody across Canada | Physical health; women's health | Self-reported chronic health conditions (back pain, head injury, HCV, asthma) | Incarcerated; pre-COVID-19 |
| 120 | Norris E, et al. (2026), Womens Health | Original research — qualitative description | Women in provincial/territorial custody in Ontario | Sexual and reproductive health; women's health | Access to desired and appropriate contraception at intake, during custody and at discharge | Other; post-COVID-19 |
| 121 | Oliffe JL, et al. (2018), Am J Mens Health | Original research — qualitative | Men transitioning into and out of a federal correctional facility in British Columbia | Mental health | Masculinities and men's mental health; depression, substance use and suicidality | Other; pre-COVID-19 |
| 122 | Ortiz-Paredes D, et al. (2022), PLoS One | Original research — qualitative | People incarcerated in federal prisons across Canada | Public health and infection control; physical health | Reasons for COVID-19 vaccine refusal; risk perception and institutional mistrust | Incarcerated; during COVID-19 |
| 123 | Owusu-Bempah A, et al. (2014), BMC Public Health | Original research — cross-sectional analysis | Indigenous and non-Indigenous adults in federal and provincial/territorial custody in British Columbia | Indigenous health | Years of life lost to incarceration, by Indigenous identity and sex | Incarcerated; pre-COVID-19 |
| 124 | Owusu-Bempah A, et al. (2023), Prev Med | Original research — population-based cohort | Black people who experienced provincial/territorial incarceration in Ontario, 2015–2020 | Substance use; mortality | Absolute and relative opioid toxicity mortality in custody and after release | Incarcerated; during COVID-19 |
| 125 | Parent S, et al. (2023), Health Promot Chronic Dis Prev Can | Original research — descriptive epidemiological study | People who died of opioid overdose in Kingston, Frontenac, Lennox and Addington, Ontario | Substance use; mortality | Characteristics of opioid overdose deaths, including deaths in correctional facilities | Incarcerated; post-COVID-19 |
| 126 | Paynter MJ, Snelgrove-Clarke E (2017), J Hum Lact | Original research — practice report | Women accessing a breastfeeding service programme in Nova Scotia (provincial/territorial) | Women's health | Breastfeeding support, prenatal education access and mother–child programme eligibility | Not incarcerated; pre-COVID-19 |
| 127 | Paynter MJ (2018), J Hum Lact | Report — policy and legal analysis | Incarcerated women who are breastfeeding in Canada | Women's health; sexual and reproductive health | Policy and legal protection for breastfeeding; correctional legislation, police lockup protocols and mother–child programs | Other; pre-COVID-19 |
| 128 | Paynter MJ, Snelgrove-Clarke E (2019), Int Breastfeed J | Commentary | Women in federal and provincial/territorial custody across Canada | Women's health | Breastfeeding support, gendered surveillance and geographic dispersion | Incarcerated; pre-COVID-19 |
| 129 | Paynter M, et al. (2021), Int J Prison Health | Original research — facility inventory and geospatial analysis | 72 facilities across Canada designated to incarcerate women and girls (federal and provincial/territorial) | Women's health | Health service responsibility by jurisdiction; distance to maternity services and mother–child programmes | Other; pre-COVID-19 |
| 130 | Paynter M, et al. (2021), J Adv Nurs | Original research — qualitative | Mothers in a provincial/territorial prison in Nova Scotia | Women's health | Experiences of maternal incarceration; family separation, visits and service denial | Other; during COVID-19 |
| 131 | Paynter M, et al. (2022), Can J Nurs Res | Review — scoping review | People incarcerated in prisons for women in Canada | Women's health; sexual and reproductive health | HIV, Pap and STI testing, contraception, pregnancy, birth outcomes and sexual assault | Other; pre-COVID-19 |
| 132 | Paynter MJ, Norman WV (2022), Semin Reprod Med | Review | People in prisons across Canada seeking abortion | Sexual and reproductive health; women's health | Barriers to abortion access: distance, cost, knowledge and institutional scheduling | Incarcerated; during COVID-19 |
| 133 | Paynter M, Heggie C (2023), Contraception | Original research — geospatial analysis | 67 institutions of incarceration for women and girls across Canada (federal and provincial/territorial) | Sexual and reproductive health; women's health | Distance from institutions to procedural abortion facilities | Incarcerated; post-COVID-19 |
| 134 | Paynter M, et al. (2023), PLoS One | Original research — qualitative case study | Pregnant people and new parents in federal prisons for women across Canada | Sexual and reproductive health | Experiences of pregnancy and early parenting; access to the institutional Mother-Child Program | Incarcerated; during COVID-19 |
| 135 | Paynter MJ, et al. (2025), BMC Womens Health | Original research — qualitative | Women and gender-diverse people who have experienced federal or provincial/territorial incarceration across Canada | Women's health; sexual and reproductive health | Family planning care: competing needs, institutional barriers, mistreatment and knowledge gaps | Incarcerated; post-COVID-19 |
| 136 | Paynter M, et al. (2025), J Obstet Gynaecol Can | Original research — cross-sectional survey (n=90; 27% Indigenous) | People in the four Atlantic provincial/territorial prisons for women | Sexual and reproductive health; women's health; public health and infection control | Pregnancy and abortion history, chlamydia and hepatitis C positivity, Pap testing and mammography | Incarcerated; post-COVID-19 |
| 137 | Peternelj-Taylor C (2018), J Forensic Nurs | Commentary | People who inject drugs in federal prisons across Canada | Physical health; substance use | Introduction of prison needle and syringe programs; needle sharing and HIV/HCV transmission | Other; pre-COVID-19 |
| 138 | Poulin C, et al. (2018), Ann Epidemiol | Original research — cross-sectional | Adults in provincial/territorial correctional facilities in Quebec | Physical health | HCV prevalence and tattooing, injection and sexual risk factors | Incarcerated; pre-COVID-19 |
| 139 | Power J, et al. (2015), Int J Forensic Ment Health | Original research — cross-sectional | Federally sentenced men across Canada | Mental health | Non-suicidal self-injury: prevalence, initiation, motivations and precipitating events | Incarcerated; pre-COVID-19 |
| 140 | Ray I, et al. (2022), Crim Behav Ment Health | Original research — cohort comparison | Men and women on remand in Ontario referred to correctional mental health services (provincial/territorial) | Mental health | Clinical, social and demographic differences by sex; psychosis, depression and self-harm | Incarcerated; during COVID-19 |
| 141 | Reekie A, et al. (2022), J Correct Health Care | Original research — cross-sectional retrospective evaluation | Admissions to a short-term provincial/territorial correctional facility in Alberta | Physical health | Opt-out STI screening uptake, case finding and treatment completion versus opt-in | Incarcerated; during COVID-19 |
| 142 | Ricciardelli R, et al. (2021), FACETS | Report — policy briefing | People in federal and provincial/territorial custody, people serving community sentences, and correctional staff across Canada | Mental health; public health and infection control; Indigenous health | COVID-19 management in custody; decarceration, reintegration and recommendations for correctional services | Other; during COVID-19 |
| 143 | Romanchuk K, et al. (2022), CMAJ Open | Original research — cross-sectional | People incarcerated in three federal prisons across Canada | Physical health; health care use | Willingness to receive SARS-CoV-2 vaccination and associated determinants | Incarcerated; during COVID-19 |
| 144 | Russell C, et al. (2022), BMC Public Health | Original research — mixed methods longitudinal follow-up | Federally incarcerated people with opioid use disorder in Ontario | Substance use; treatment | Opioid agonist treatment experiences in custody and release planning | Incarcerated; pre-COVID-19 |
| 145 | Ryan C, et al. (2020), Can J Public Health | Commentary | Incarcerated Indigenous women in correctional facilities across Canada | Physical health; mental health; Indigenous health; women's health | Public health and human rights implications of COVID-19; physical distancing and stress | Incarcerated; during COVID-19 |
| 146 | Shaw J, Driftmier P (2022), OMEGA J Death Dying | Original research — qualitative (n=9) | Men serving life sentences in a federal penitentiary in Canada | Mortality; physical health | Perspectives on end-of-life care and medical assistance in dying in custody | Incarcerated; pre-COVID-19 |
| 147 | Simpson AIF, et al. (2023), Med Sci Law | Other — editorial | People with mental disorder in the prison population across Canada | Mental health; mortality | Ethical considerations regarding medical assistance in dying; suicide risk and equivalence of care | Incarcerated; during COVID-19 |
| 148 | Singh D, et al. (2019), CMAJ | Commentary | Indigenous people in federal and provincial/territorial custody in British Columbia | Indigenous health | Years of life lost to incarceration for Indigenous men and women | Incarcerated; pre-COVID-19 |
| 149 | Slaunwhite AK, et al. (2026), BC Med J | Original research — linked administrative data (20% random sample) | People in provincial/territorial custody in British Columbia vs the non-incarcerated BC population, 2021 | Mental health; substance use; social determinants of health | Prevalence of mental illness, substance use disorder, co-occurring disorders, income assistance and no fixed address | Incarcerated; during COVID-19 |
| 150 | Stewart LA, Wilton G (2014), Crim Justice Stud | Original research — cohort study | Federally sentenced adults with mental disorders across Canada | Mental health | Distribution of mental disorders and correctional outcomes by sex and Indigenous identity | Incarcerated; pre-COVID-19 |
| 151 | Stewart LA, et al. (2016), Int J Law Psychiatry | Original research — cross-sectional (n=488) | Men entering federal custody across Canada | Mental health | Prevalence and profile of cognitive deficits and correctional outcomes | Incarcerated; pre-COVID-19 |
| 152 | Stewart LA, et al. (2018), Int J Prison Health | Original research — cross-sectional | Federally sentenced adults across Canada | Social determinants of health; physical health | Relationship between social determinants and self-reported physical health conditions at admission | Incarcerated; pre-COVID-19 |
| 153 | Struthers Montford K (2022), Soc Leg Stud | Original research — qualitative | People in provincial/territorial prisons in Ontario | Physical health | Prison food quality under cook-chill provision; loss of dietary autonomy | Incarcerated; during COVID-19 |
| 154 | Subramanian Y, et al. (2016), Int J Prison Health | Original research — retrospective cohort | People living with HIV at a provincial/territorial remand centre in Alberta | Physical health | Antiretroviral adherence, virologic suppression and retention in community care after release | Incarcerated; pre-COVID-19 |
| 155 | Taylor S, et al. (2021), Health Justice | Review | Justice-involved women in Canada | Physical health | Mobility and vulnerability to sexually transmitted and blood-borne infections | Incarcerated; during COVID-19 |
| 156 | van der Meulen E (2017), Subst Use Misuse | Original research — qualitative | Former federal incarcerated people across Canada who injected drugs | Substance use | In-prison injection drug use, drug availability and the case for needle and syringe programs | Incarcerated; pre-COVID-19 |
| 157 | van der Meulen E, et al. (2017), Women Crim Justice | Original research — qualitative | Women with federal incarceration experience across Canada | Substance use | Effects of punitive drug policy; access to harm reduction supplies and culturally relevant programming | Not incarcerated; pre-COVID-19 |
| 158 | van der Meulen E, et al. (2017), Prison J | Original research — qualitative (focus groups and interviews, n=30) | Former federal incarcerated people in Ontario | Substance use; public health and infection control | Views on prison needle and syringe programs; in-prison injection, needle sharing and program design | Not incarcerated; pre-COVID-19 |
| 159 | Vaughan AD, et al. (2017), J Forensic Leg Med | Original research — cross-sectional analysis of administrative records | Men who died in custodial settings (correctional and police) in Ontario (federal and provincial/territorial) | Mental health; substance use | In-custody deaths among people with mental illness or substance use disorder, by setting | Not reported; pre-COVID-19 |
| 160 | Waldner D, et al. (2021), FACETS | Review | Canadian population in the pre-vaccine pandemic era, including people in custody | Physical health | COVID-19 case numbers, risk factors and outbreak drivers in correctional facilities | Incarcerated; during COVID-19 |
| 161 | Webster P (2015), CMAJ | Commentary | People held in segregation or seclusion in prisons across Canada | Mental health | Case for prohibiting segregation for people who are suicidal, self-harming or seriously mentally ill | Incarcerated; pre-COVID-19 |
| 162 | Whitten D, et al. (2023), J Assoc Med Microbiol Infect Dis Can | Original research — retrospective review | Admissions to provincial/territorial correctional facilities in Newfoundland and Labrador | Physical health; sexual and reproductive health | Rates of sexually transmitted and blood-borne infection testing at admission | Incarcerated; during COVID-19 |
| 163 | Whittingham L, et al. (2020), J Appl Res Intellect Disabil | Original research — retrospective cohort (linked administrative data) | Adults with developmental disabilities released from provincial/territorial prisons in Ontario vs the general population | Mental health | Prevalence and health status of people with developmental disabilities | Not incarcerated; pre-COVID-19 |
| 164 | Wilson A (2023), Crit Criminol | Original research — qualitative document and narrative analysis | People in federal prisons across Canada under the Food Services Modernization Initiative | Physical health | Food quality, nutritional adequacy and contested institutional narratives about food | Other; post-COVID-19 |
| 165 | Wilton G, Stewart LA (2017), Psychiatr Serv | Original research — cohort study | Federally sentenced adults with co-occurring disorders across Canada | Mental health; substance use | Correctional outcomes associated with mental disorder, substance use disorder and both | Incarcerated; during COVID-19 |
| 166 | Zendo S, et al. (2021), Soc Work Public Health | Original research — qualitative narrative study | Women in provincial/territorial correctional institutions in Ontario | Women's health | Experiences accessing health services; segregation, socioeconomic status and mental health | Incarcerated; during COVID-19 |
| 167 | Zygmunt A, et al. (2024), Int J Prison Health | Original research — retrospective epidemiological analysis | People incarcerated and staff in provincial/territorial correctional facilities in Ontario, 2020–2022 | Physical health; public health and infection control | COVID-19 case burden in correctional versus non-correctional populations | Incarcerated; post-COVID-19 |
